# Functional Analysis of Free Fatty Acid Receptor GPR120 in Human Eosinophils: Implications in Metabolic Homeostasis

**DOI:** 10.1371/journal.pone.0120386

**Published:** 2015-03-19

**Authors:** Yasunori Konno, Shigeharu Ueki, Masahide Takeda, Yoshiki Kobayashi, Mami Tamaki, Yuki Moritoki, Hajime Oyamada, Masamichi Itoga, Hiroyuki Kayaba, Ayumi Omokawa, Makoto Hirokawa

**Affiliations:** 1 Department of General Internal Medicine and Clinical Laboratory Medicine, Akita University Graduate School of Medicine, Akita, Japan; 2 Department of Otolaryngology, Kansai Medical University, Shin-machi, Hirakata City, Osaka, Japan; 3 Department of Clinical Laboratory Medicine, Hirosaki University Graduate School of Medicine, Hirosaki, Japan; Universidade Federal do Rio de Janeiro, BRAZIL

## Abstract

Recent evidence has shown that eosinophils play an important role in metabolic homeostasis through Th2 cytokine production. GPR120 (FFA4) is a G protein-coupled receptor (GPCR) for long-chain fatty acids that functions as a regulator of physiological energy metabolism. In the present study, we aimed to investigate whether human eosinophils express GPR120 and, if present, whether it possesses a functional capacity on eosinophils. Eosinophils isolated from peripheral venous blood expressed GPR120 at both the mRNA and protein levels. Stimulation with a synthetic GPR120 agonist, GW9508, induced rapid down-regulation of cell surface expression of GPR120, suggesting ligand-dependent receptor internalization. Although GPR120 activation did not induce eosinophil chemotactic response and degranulation, we found that GW9508 inhibited eosinophil spontaneous apoptosis and Fas receptor expression. The anti-apoptotic effect was attenuated by phosphoinositide 3-kinase (PI3K) inhibitors and was associated with inhibition of caspase-3 activity. Eosinophil response investigated using ELISpot assay indicated that stimulation with a GPR120 agonist induced IL-4 secretion. These findings demonstrate the novel functional properties of fatty acid sensor GPR120 on human eosinophils and indicate the previously unrecognized link between nutrient metabolism and the immune system.

## Introduction

Eosinophils are generally found in low numbers within the circulation, while the majority of eosinophils at baseline reside within mucosal tissues interfacing with the environment and within primary and secondary lymphoid tissues [1]. The gastrointestinal tract, lungs, and skin are the principal sites of accumulation [2,3]. Once eosinophils leave the circulation, their longevity is enhanced in these tissues, where they play a central beneficial role in the clearance of parasitic and other infections, primarily through the release of toxic granule proteins. In addition, eosinophils also reside in visceral adipose tissue under noninflammatory conditions and help maintain metabolic homeostasis and glucose tolerance through Th2 cytokine-dependent regulation of macrophage activity [4–6]. For instance, a recent study has indicated that exercise triggers eosinophil secretion of IL-4, which is indispensable for macrophage differentiation and thermogenesis in adipose tissue [7]. Thus, eosinophils are multifunctional leuckocytes involved not only in allergic diseases and innate immunity but also in physiological regulation of energy metabolism as an important source of Th2 cytokines.

GPR120 (also called FFA4), a member of the rhodopsin-like family of G protein-coupled receptors (GPCRs), is highly conserved across many species [8]. Hirasawa *et al*. recently deorphanized GPR120 that is activated by a series of long-chain free fatty acids (FFAs) [9]. GPR120 is abundantly expressed in the intestine, and its stimulation causes incretin hormone glucagon peptide-1 secretion. GPR120 stimulation with natural and synthetic agonists inhibits the secretion of inflammatory cytokines in monocytes and macrophages, resulting in improvement of insulin resistance in obesity [10]. GPR120-deficient mice fed on a high-fat diet develop obesity, glucose intolerance, and fatty liver with decreased adipocyte differentiation [11]. Despite accumulating studies describing the roles of GPR120 in association with physiological energy metabolism, little is known in terms of eosinophil functions. In this study, we aimed to investigate whether human eosinophils express GPR120 and, if present, whether it possesses a functional capacity on eosinophils.

## Materials and Methods

### Cell preparation

Peripheral venous blood was obtained from non-obese subjects with mild eosinophilia (approximately 4–8% of total white blood cells). None of them were being treated with any medication, including systemic anti-allergic agents. Informed written consent was obtained from each subject, and the study protocol was approved by the Ethics Committee of Akita University School of Medicine. Peripheral blood mononuclear cells (PBMCs) were isolated from peripheral venous blood by Ficoll-Paque (Pharmacia, Uppsala, Sweden) density gradient centrifugation, and monocytes were purified using a monocyte isolation kit and a MACS system (Miltenyi Biotec) [12]. Peripheral blood granulocytes were isolated by sedimentation with 6% dextran followed by centrifugation on 1.088 Percoll (Pharmacia) density gradients. Eosinophils were isolated from granulocyte pellets by negative selection using anti-CD16 immunomagnetic beads and a MACS system (Miltenyi Biotec, Bergisch Gladbach, Germany) as previously described [13,14]. Eosinophil purity of >98% was routinely obtained as determined by microscopic analyses.

### RT-PCR

Total RNA was extracted using Isogen (Nippongene, Toyama, Japan) and reverse-transcribed with the Transcriptor First Strand cDNA Synthesis Kit (Roche Applied Science, Tokyo, Japan). One microliter of the cDNA synthesis reaction was used as a template for PCR amplification with the FastStart High Fidelity PCR System (Roche Applied Science, Tokyo, Japan). The following primers were used: for human GPR120, the forward primer was 5’-TGGAGATGCACATTGTTTGGAGA-3’, reverse: 5’-AGCCTCCAAGTGGTGGAGTGA-3’ (GenBank accession number: NM001195755); for β-actin, the forward primer was 5’-TGGCACCCAGCACAATGAA-3’, and the reverse primer was 5’-CTAAGTCATAGTCCGCCTAGAAGCA-3’ (GenBank accession number: NM001101). The expected size of the amplified product was 130 bp (GPR120) and 189 bp (β-actin). After the initial denaturation at 95°C for 10 minutes, 45 cycles of denaturation for 10 seconds at 95°C, annealing for 10 seconds at 60°C, and extension for 25 seconds at 72°C were carried out. All reactions were conducted using the C1000 Thermal Cycler (Bio-Rad Laboratories, Inc., Hercules, CA). The amplified products were separated by electrophoresis on 12.5% polyacrylamide gel (GE Healthcare Japan, Tokyo, Japan), and the gel was subjected to silver staining.

### Immunocytochemical staining for GPR120

Eosinophils, seeded in 8-well Millicell EZ slides (Millipore, Billerica, MA), were incubated for 5 min in 0.1% bovine serum albumin (BSA) and phorbol 12-myristate 13-acetate (1 ng/ml, Sigma-Aldrich, St Louis, MO) to adhere to the slide and then fixed with 3% paraformaldehyde for 10 min. The slides were washed with phosphate buffered saline (PBS), incubated with blocking buffer (1% BSA PBS) overnight, and incubated with rabbit GPR120 antibody (1:100, Bioworld Technology, Inc., St. Louis Park, MN) or normal rabbit IgG antibody (Santa Cruz Biotechnology, Santa Cruz, CA) for 120 min. Next, the slides were incubated with Alexa Fluor 488 goat anti-rabbit IgG (1:200, Life Technologies Corporation) for 30 min. To visualize nuclei, slides were stained with Hoechst 33342 trihydrochloride trihydrate (Life Technologies Corporation). The slides were analyzed using a confocal microscope (LSM510; Carl Zeiss Co., Ltd, Jena, Germany).

### Flow cytometric analysis for receptor expression

For GPR120 internalization experiments, eosinophils were resuspended at 1.0 × 10^6^ cells/ml in RPMI 1640 medium (Life Technologies Corporation, Carlsbad, CA) with 3% fetal bovine serum (FBS), and incubated with or without the indicated concentrations of GPR120 agonist GW9508 (Cayman Chemical, Ann Arbor, MI) at 37°C in humidified air with 5% CO_2_ for the indicated time interval. Cells were then stained with anti-human GPR120 mAb (1:50, Bioworld Technology, Inc.) or isotype-matched control mAb (Santa Cruz Biotechnology) for 30 min on ice. Next, the cells were incubated with Alexa Fluor 488 goat anti-rabbit IgG (1:100, Life Technologies Corporation) for 30 min on ice. To study Fas (CD95) expression, eosinophils were treated with or without 100 μM of GW9508 for 24 h, after which cells were stained with fluorescein isothiocyanate (FITC)-conjugate anti-human CD95 mAb (1:50, mouse IgG; Beckman Coulter, Miami, FL) or isotype-matched control mAb (Beckman Coulter) for 30 min at 4°C. To investigate the expression of GPR40, cells were fixed and permeabilized with BD cytofix/cytoperm (BD Bioscience, San Jose, CA), and then stained with anti-human GPR40 mAb (1:50, Gene Tex, Inc., Irvine, CA) or isotype-matched control mAb (Santa Cruz Biotechnology) for 30 min. The cells were incubated with Alexa Fluor 488 goat anti-rabbit IgG (1:100, Life Technologies Corporation) for 30 min. According to the manufacturer’s instructions, the HeLa cell line was used as a positive control for GPR40. The cells were analyzed using a flow cytometer (FACScan, Becton Dickinson Immunocytometry Systems, San Jose, CA). Data were analyzed by Flowjo (Ver. 9.2, Tree Star, Ashland, OR). The expression of GPR120 was assessed as the ratio of the mean fluorescence intensity (MFI) of the sample and the isotype-matched control.

### Chemotaxis assay

Chemotaxis assay was conducted in duplicate using 5-μm-pore polycarbonate, polyvinylpyrrolidone-free membranes in Boyden chambers [15]. Aliquots of 100 μl of the cell suspension at 0.5 × 10^6^ cells/ml were placed in the upper chambers. GW9508 or human eotaxin-1 (CCL11; 10 nM, R&D Systems, Minneapolis, MN) was placed in the lower chambers. To study the effect of GW9508 on eotaxin-induced chemotaxis, eosinophils were preincubated with GW9508 at the indicated concentration for 1 h at 37°C, and the cells were washed once. Cell viability did not differ under each condition. The loaded chambers were incubated at 37°C in humidified air with 5% CO_2_ for 1 h. Then, the membrane was removed, followed by fixation and staining for 3 min in May-Grünwald solution. The cells that migrated and adhered to the lower surface of the membrane were counted from 10 fields by light microscopy.

### Degranulation assay

A 96-well plate was coated with 3% human serum albumin in HBSS for 2 h at 37°C and washed three times with HBSS before use. Purified eosinophils were suspended in RPMI 1640 with 0.1% HSA in the 96-well plate. Cells were then incubated with the indicated concentrations of GW9508 for 4 h under the same conditions [16,17]. The supernatants were separated by centrifugation, and the concentration of eosinophil-derived neurotoxin (EDN) was measured using a EDN ELISA kit (Medial Biological Laboratories, Nagoya, Japan) according to the manufacturer’s instructions.

### Determination of cell apoptosis

Eosinophils were resuspended at 1.0 × 10^6^ cells/ml in RPMI 1640 with 10% FBS, and incubated with or without the indicated concentrations of GW9508 at 37°C in humidified air with 5% CO_2_ for the indicated time interval. To study the involvement of GPR40, eosinophils were preincubated with GW1100 (10 μM, Cayman chemical)[18] or vehicle for 30 min, followed by treatment with GW9508 for 48 h. Phosphoinositide 3-kinase (PI3K) inhibitors (1 μM of LY294002; pan-PI3K inhibitor, 0.1 μM AS605240; PI3Kγ selective inhibitor: Cayman chemical) were added to culture medium 10 min prior to stimulation with 100 μM of GW9508. The concentration of PI3K inhibitors used was based on previous studies [19,20]. An apoptosis detection kit (Medial Biological Laboratories) was used to quantitatively determine eosinophils undergoing apoptosis by virtue of their ability to bind to annexin V and propidium iodide (PI). Briefly, harvested eosinophils were washed in cold PBS and stained with annexin V and PI according to the manufacturer’s instructions. The stained cells were analyzed using a FACScan flow cytometer. In some experiments, eosinophils were incubated with 1 ng/ml of IL-5 (R&D Systems).

### Measurement of caspase-3 activity

Eosinophils were resuspended at 1.0 × 10^6^ cells/ml in RPMI 1640 medium with 10% FBS, and incubated with or without 100 μM of GW9508 for 48 h. Caspase-3 activity in these cells was assayed with an APOPCYTO colorimetric assay kit (Medial Biological Laboratories) according to the manufacturer’s instructions.

### Determination of IL-4 release from eosinophils

ELISpot was performed on human eosinophils using an IL-4 ELISpotpro kit (Mabtech, Nacka Strand, Sweden). Cells were seeded at 2.5 × 10^6^ cells/ml in RPMI 1640 with 0.5% FBS, and incubated with or without the indicated concentrations of GW9508 at 37°C in humidified air with 5% CO_2_ for 18 h. Calcium ionophre A23187 (Sigma-Aldrich) was used as a positive control [21]. To study the involvement of GPR40, eosinophils were preincubated with GW1100 (10 μM) or vehicle for 30 min, followed by treatment with GW9508 for 18 h. Assay was conducted according to the manufacturer’s instructions. Developed spots were counted under stereoscopic microscope (Leica MZ16 F, Leica Microsystems, Wetzlar, Germany). All scoring was performed by a single investigator in a coded manner.

### Statistical analysis

Data are presented as means ± SEMs. Comparisons of two groups of data were performed using the Student’s paired t test or Wilcoxon signed rank test. One-way ANOVA with repeated measures was used for comparison of more than two variables. When the initial p value was less than 0.05, Bonferroni-Dunn post-hoc test was used to determine the significance between groups. The data were analyzed with GraphPad Prism version 5.04 software (GraphPad Software, San Diego, CA). Significance was established at the p<0.05 level.

## Results

### Expression of GPR120 on human eosinophils

**Fig 1:**
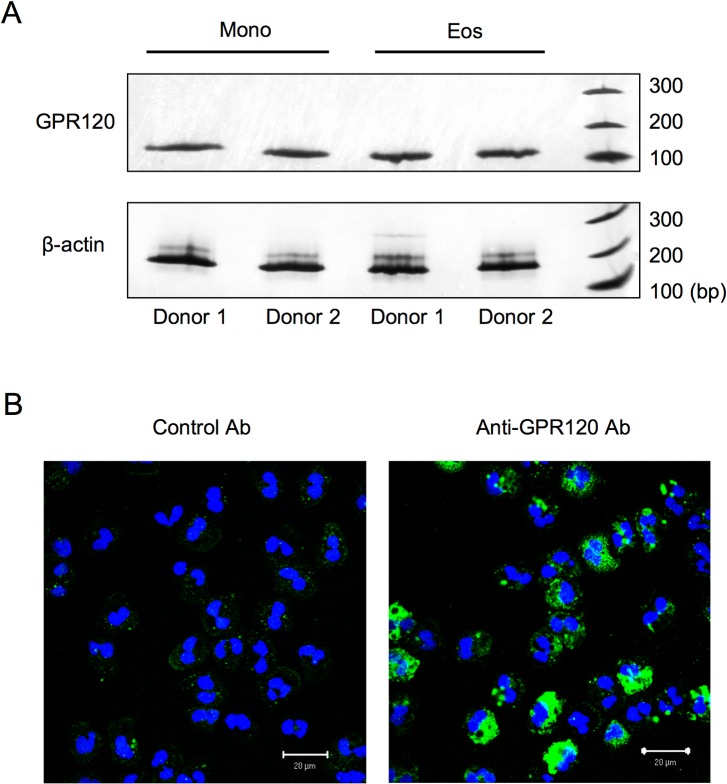
Expression of GPR120 in isolated human eosinophils. (A) The gene expression profile in isolated human eosinophils and monocytes was studied using RT-PCR. The amplified products were separated on a 12.5% polyacrylamide gel, and the gel was subjected to silver staining. GPR120 mRNA (130 bp) is expressed in eosinophils. As a housekeeping gene, β-actin (189 bp) was used for the loading control. (B) Nonpermeabilized eosinophils were stained with anti-GPR120 antibody or isotype-matched control antibody (green), and then subjected to confocal microscopy with identical settings. To visualize nuclei, slides were stained with Hoechst 33342 (blue). The results are representative of two independent donors with similar results.

Although the expression of GPR120 had been determined on monocytes/macrophages [10], its expression on human blood eosinophils is yet to be determined. First, peripheral blood eosinophils and monocytes were isolated, and expression of GPR120 was investigated using RT-PCR. As shown in Fig. 1A, similar to monocytes, eosinophils expressed GPR120 mRNA. Surface protein expression on purified eosinophils was confirmed by immunocytochemical staining using GPR120 Ab (Fig. 1B).

### Ligand stimulation induces down-regulation of GPR120 on eosinophil surface

**Fig 2:**
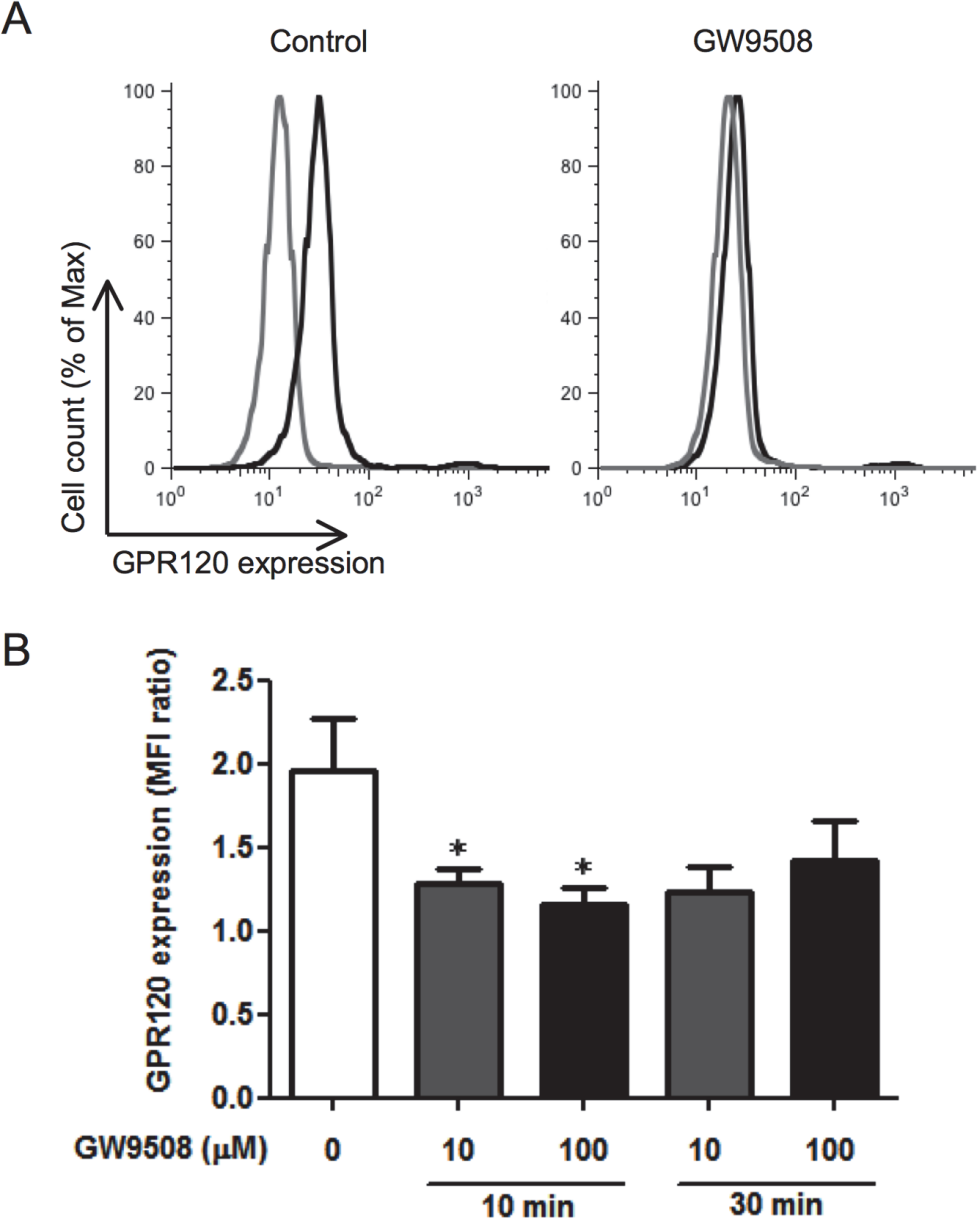
Down-regulation of GPR120 in human eosinophils by ligand stimulation. (A) After incubation with GW9508 (100 μM) for 10 min, nonpermeabilized eosinophils were stained with anti-GPR120 antibody (black histogram) or isotype-matched control (gray histogram) and then subjected to flow cytometric analysis. The histograms indicated monomodal expression of GPR120 and ligand-induced down-regulation. One of five experiments from different donors is shown. (B) After incubation with the indicated concentration of GW9508 for 10 or 30 min, the expression of GPR120 on the eosinophil surface was assessed as the MFI ratio. Data are expressed as the mean of five experiments ± SEM from different donors. *p<0.05 vs non-stimulated cells.

The synthetic agonist GW9508 is widely used to explore the biology of GPR120. However, GW9508 is not specific for GPR120 and is also stimulated another FFA receptor GPR40 (FFA1) [18]; therefore, we assessed the GPR40 expression on human eosinophils by flow cytometry. In contrast to Hela cells used as a positive control, the expression of GPR40 on eosinophils was negligible (A in S1 Fig.). Given this result, GW9508 was used as a functional GPR120-specific agonist in eosinophils. We first investigated the surface receptor expression level using flow cytometry followed by stimulation with GW9508 since GPR120 has been shown to translocate from the plasma membrane to the cytosol rapidly after ligand stimulation [9,10]. As expected, 10 min of ligand stimulation induced down-regulation of GPR120 (Fig. 2A, B), although the effect was not statistically significant at 30 min of stimulation due to variation in each experiment (Fig. 2B).

### GPR120 agonist has no effect on eosinophil chemotaxis and degranulation

Eosinophils express a variety of GPCRs, many of which are associated with chemotaxis. To investigate the functional roles of GPR120 on human eosinophils, we initially tested migration toward GW9508 using a Boyden chamber system, but GW9508 itself showed no chemotactic response (A in S2 Fig.). Next, the capability to modulate eosinophil chemotaxis toward eotaxin-1 (CCL11), which plays an indispensable role in eosinophilic inflammation, was examined. However, GW9508 treatment did not affect eotaxin-induced eosinophil migration (B in S2 Fig.). To evaluate the effect on granule protein release, we measured the concentrations of eosinophil-derived neurotoxin (EDN) in culture supernatant after stimulation with GW9508. As shown in C in S2 Fig., no significant effect was observed as a result of agonist stimulation. These results indicated that GPR120 activation did not affect eosinophil chemotaxis and degranulation.

### GPR120 agonist induces eosinophil survival by inhibiting spontaneous apoptosis

**Fig 3:**
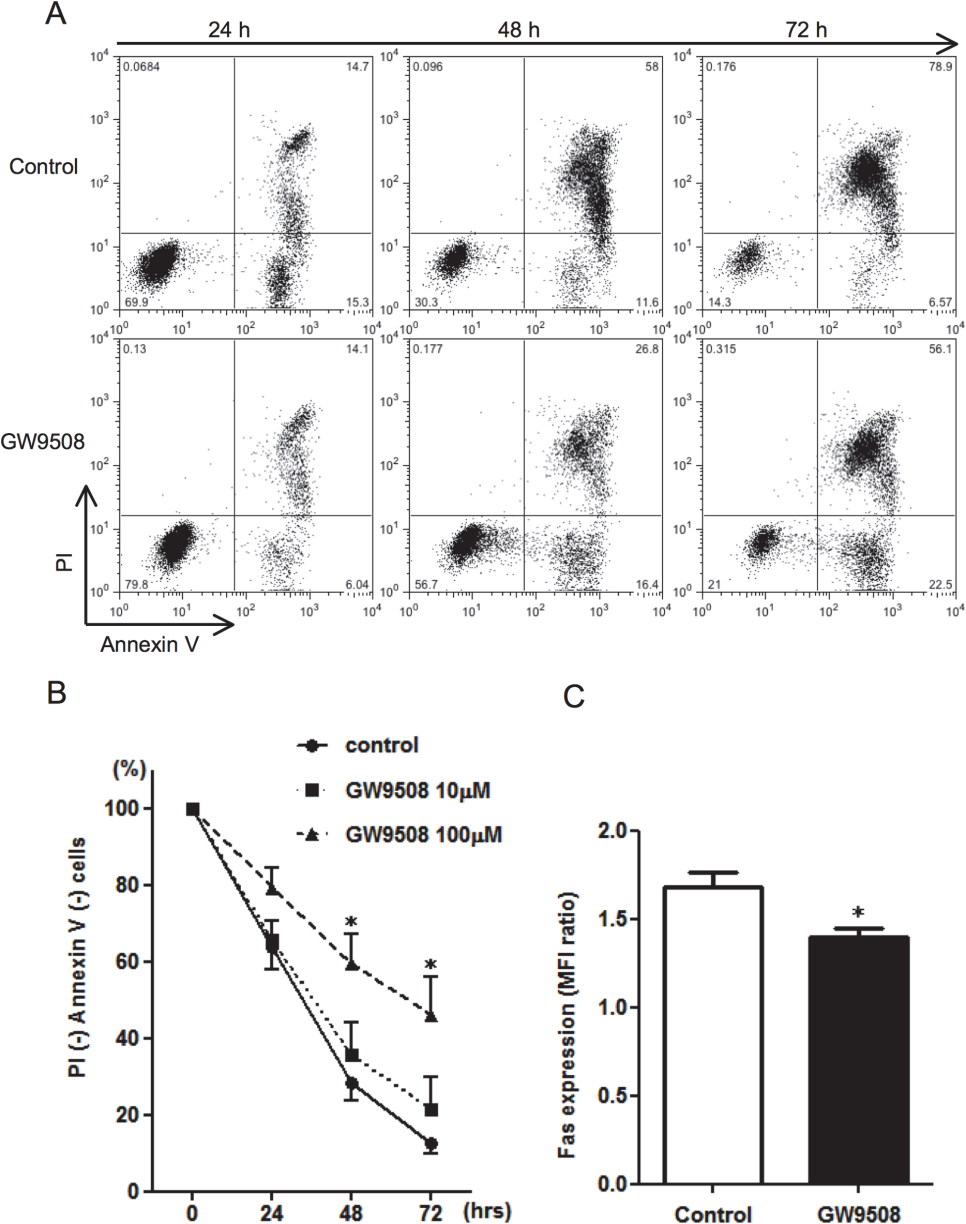
The effect of GPR120 agonist on eosinophil spontaneous apoptosis. (A) To examine the capacity to modulate eosinophil survival, purified eosinophils were incubated with or without GW9508 (100 μM) for the indicated times. Eosinophil apoptosis was then determined by flow cytometry by staining with Annexin V and propidium iodine (PI). The data show one representative with similar results, indicating the percentage of the total cells in each quadrant. (B) Time course and concentration-dependent response of eosinophil survival. Eosinophil viability was assessed by the percentage of Annexin V (-) and PI (-) cells. Data are expressed as the mean of six experiments ± SEM from different donors. *p<0.05 vs control. (C) The effect of GW9508 on Fas receptor expression. Eosinophils were treated with or without GW9508 (100 μM) for 24 h, after which the expression of Fas receptors on the eosinophil surface was assessed as the MFI ratio using a flow cytometer. Data are expressed as the mean of four experiments ± SEM from different donors. *p<0.05 vs control.

Next, we examined the capacity to modulate eosinophil apoptosis that spontaneously occurs after culturing these cells. Purified eosinophils were incubated with GW9508 or vehicle control for up to 72 h. Eosinophil apoptosis was determined by flow cytometry; Annexin V was used to stain the early phase apoptotic cells, while PI was used to stain the late phase cells. As shown in Fig. 3A and B, approximately 70% of cells underwent spontaneous apoptosis after 48 h, although GW9508 (100 μM) significantly inhibited the apoptosis and increased the percentage of live cells (annexin and PI both negative). After culturing cells for 72 h, live cells in the control were 12.7 ± 2.6%, whereas GW9508-stimulated cells were 46.1 ± 9.9%. To confirm that prolonged eosinophil survival was not mediated through GPR40, GPR40 blockade was also examined. GW1100, a specific antagonist for GPR40 reported as being without effect on GPR120 [18], did not affect the anti-apoptotic effect of GW9508 (B in S1 Fig.). Since IL-5 is a well-known eosinophil survival factor, we also examined whether GW9508 enhances IL-5-induced eosinophil survival. In the presence of IL-5 (1 ng/ml), the effect of GW9508 on cell viability was not observed at 48 h (live cells in control and GW9508-stimulated cells; 87.6 ± 2.9 and 88.4 ± 3.3%, respectively, n = 6) and 72 h (82.9 ± 6.4 and 80.6 ± 7.3%, respectively, n = 6) of incubation. Lower concentrations of GW9508 (1 and 10 μM) had no significant effect on IL-5-induced eosinophil survival (data not shown).

Cytokine-deprived eosinophils exhibited a time-dependent increase in Fas (CD95) expression that mediates eosinophil apoptosis [22,23]. To obtain multiple lines of evidence that indicate the anti-apoptotic effect of GW9508, we examined whether GW9508 is associated with down-regulation of the cell surface Fas expression. As expected, stimulating eosinophil with GW9508 for 24 h resulted in a decrease in Fas expression compared to non-stimulated control (Fig. 3C).

### Involvement of PI3K and caspase-3 in GPR120 agonist induced anti-apoptotic effect

**Fig 4:**
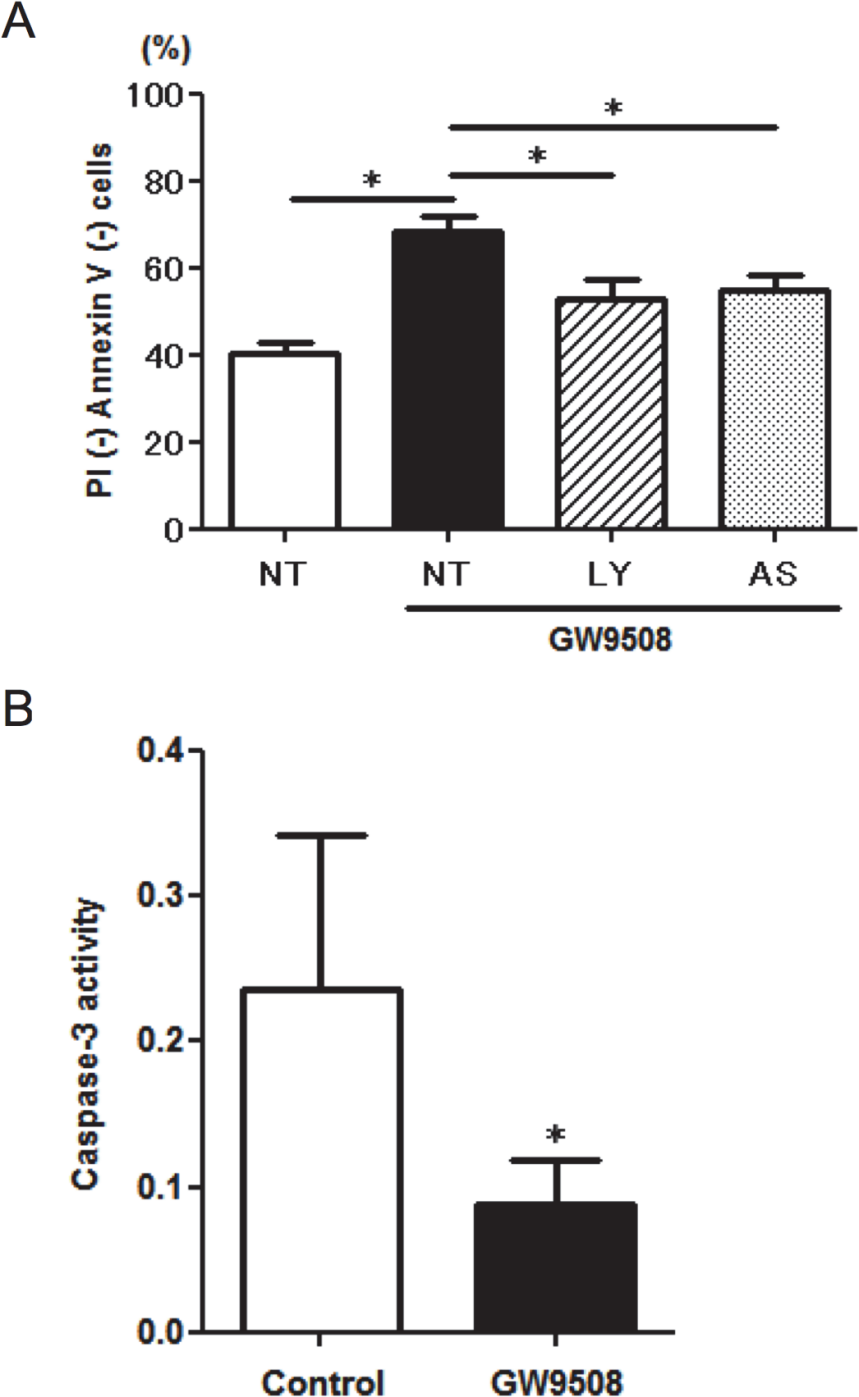
The GPR120 agonist-induced anti-apoptotic effect was inhibited by PI3K inhibitors and was associated with decreased caspase-3 activity. (A) The effect of GW9508 on PI3K pathway. After preincubation with a PI3K inhibitor (LY294002; 1 μM, AS605240; 0.1 μM) or vehicle for 10 min, eosinophils were treated with GW9508 (100 μM) for 48 h. Apoptosis assay was conducted by the above-mentioned method. The percentage of live cells (Annexin V- and PI-negative cells) was measured and the data are expressed as the mean of four experiments ± SEM from different donors. *p<0.05 vs GW9508 alone. NT: non-treatment, LY: LY294002 (pan-PI3K inhibitor), AS: AS605240 (PI3Kγ selective inhibitor). (B) The effect of GW9508 on caspase-3 activity. Eosinophils were treated with or without GW9508 (100 μM) for 48 h, followed by measurement of the activity of caspase-3, measured colorimetrically in arbitrary units using a colorimetric assay kit. Data are expressed as the mean of five experiments ± SEM from different donors. *p<0.05 vs control.

It has been reported that GW9508 promotes PI3K pathway-dependent angiogenic activity in colorectal carcinoma cells [24]. To test whether PI3K signaling was involved in the anti-apoptotic effect, we examined the effect of PI3K inhibitors on the GW9508-induced eosinophil survival. As shown in Fig. 4A, both pan-PI3K inhibitor LY294002 and PI3Kγ-selective inhibitor AS605240 attenuated GW9508-induced eosinophil survival. As caspase-3 is the final effector caspase whose targets include the DNA nuclease responsible for the characteristic nuclear degradation of apoptosis [22], we next measured the enzymatic activity of caspase-3 by cleavage of the tetrapeptide *N*-acetyl-Asp-Glu-Val-Asp-*p*-nitroanilide using a colorimetric assay kit. As expected, a significant decrease in caspase-3 activity was observed in GW9508-treated cells (Fig. 4B). These results suggested that GPR120 activation transduced the PI3K signaling pathway and inhibited the enzymatic activity of caspase-3, which resulted in resistance to spontaneous apoptosis.

### GPR120 agonist induces IL-4 release

**Fig 5:**
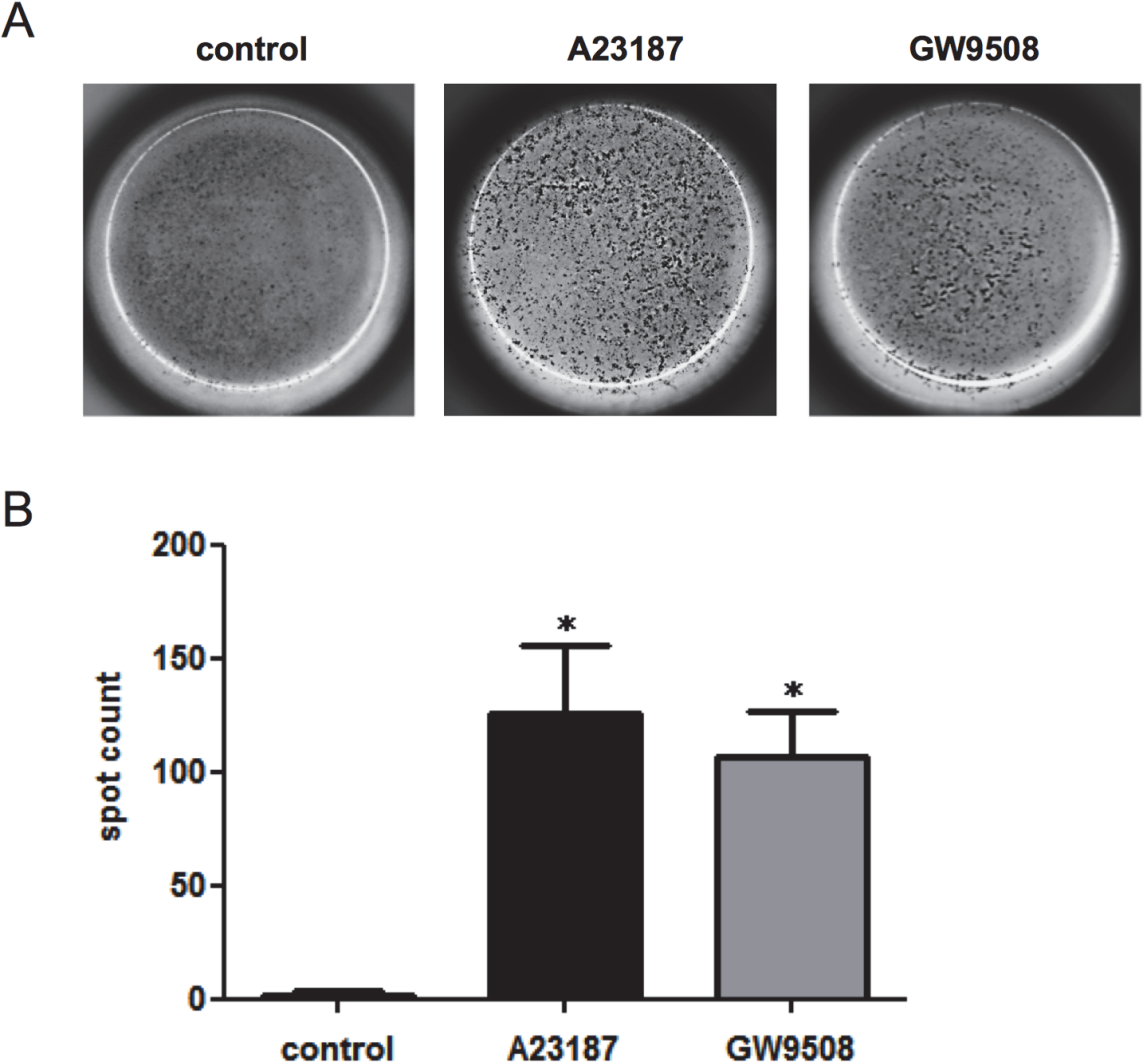
ELISpot assay for IL-4 secretion induced by GPR120 agonist. (A) IL-4 ELISpot assay was performed on eosinophils following stimulation with GW9508 (100 μM) or vehicle for 18 h. A23187 (0.5 μM) was used for a positive control. Developed spots represent the number of IL-4 secreted from human eosinophils. Image was taken by stereoscopic microscope. (B) Developed spots were counted by a single investigator in a coded manner. Data are expressed as the mean of four experiments ± SEM from different donors. *p<0.05 vs control.

Eosinophils have recently been recognized as the predominant IL-4-producing cells in adipose tissue [25]. Since IL-4 plays a critical role in metabolic homeostasis and glucose tolerance by maintaining alternative macrophages [5,7,25], we examined IL-4 secretion by ELISpot assay after stimulation with GW9508 for 18 h. As shown in Fig. 5A and B, GW9508 significantly induced IL-4 secretion from eosinophils, which was comparable with A23187 used as a positive control. To confirm that it was not mediated through GPR40, GPR40 blockade was also examined using GW1100. We confirmed that GW9508-induced IL-4 secretion was not affected by GW1100 (C in S1 Fig.).

## Discussion

FFAs are an important source of energy, and those that are polyunsaturated are essential components for the human body [26]. In addition to those metabolic roles, recent evidence has clearly indicated that they can regulate various cellular functions as bioactive ligands for GPCRs. To date, several GPCRs have been identified as FFA receptors: GPR40, GPR41, GPR43, GPR84, GPR119, and GPR120 [27]. Among them, GPR120 is attracting attention with nutrient-sensing capabilities and regulation of energy metabolism. Given that accumulating evidence links eosinophils and metabolic homeostasis, the present study was aimed to study the expression and functional capacities of GPR120 on eosinophils.

Studies using isolated human eosinophils are essential to understand eosinophil biology. Our data clearly indicated GPR120 expression on the surface of human peripheral blood eosinophils (Fig. 1). To date, significant GPR120 mRNA expression has been observed in the lungs, adipose tissue, adrenal glands, and gastrointestinal tract [9,10]. Various types of cells have been demonstrated to express GPR120: intestinal endocrine cells, adipocytes, taste cells, bone marrow-derived dendritic cells, Kupffer cells, and monocytes/macrophages [9,10,28,29]. In spite of the constitutive GPR120 expression, eosinophils did not express detectable levels of GPR40. A similar expression pattern was observed in macrophages and adipocytes [10].

Given the negligible expression of GPR40 in eosinophils, we employed a small molecule, synthetic compound GW9508 to pursue the biology of GPR120. In the present study, we did not attempt to examine the effect of FFAs for several reasons. C14 to C18 saturated or C16 to 22 mono- and polyunsaturated long-chain FFAs including docosahexsaenoic acid (DHA, 22:6) are reported to bind to GPR120 [9], although they can also interact with multiple targets, such as GPR40 and peroxisome proliferator-activated receptors (PPARs) [23,30]. PPARs and their heterodimer partner retinoid X receptors (RXRs) are constitutively expressed in human eosinophils [13,14]. Both PPARα agonists and PPARγ agonists affect eosinophil survival and eotaxin-induced chemotaxis [13,22,31]. A genetic approach is limited because isolated eosinophils are terminally differentiated, relatively short-lived, and non-dividing cells. Thus, using GW9508 was quite reasonable to study the function of GPR120.

Consistent with previous reports, rapid down-regulation of cell surface GPR120 was observed after stimulation with GW9508 (Fig. 2). Similar to other GPCRs, ligand-dependent internalization of GPR120 is a result of translocation from the plasma membrane to the cytosol. After ligand binding, recruited β-arrestin can bind to the cytosolic sites on GPR120 and mediate receptor endocytosis [10,30]. In macrophages, internalized GPR120-β-arrestin2 complexes inhibit lipopolysaccharide-mediating proinflammatory signaling cascades by sequestering a component of the signaling pathway in an inactive form [10]. In contrast to the critical roles of β-arrestin2 in the anti-inflammatory effects in macrophages, β-arrestin2 was not required for enhancement of glucose uptake in adipocytes [10]. Our results indicate that rapid ligand-dependent internalization is also occurring in eosinophils.

Among various GPCRs expressed by eosinophils, CCR3 plays an indispensable role in eosinophil-specific accumulation in interaction with eotaxins. Since GW9508 itself did not exhibit direct chemotactic activity, eosinophils were exposed to GW9508 and then eotaxin-directed chemotaxis assay was performed. However, GW9508 did not enhance or attenuate chemotactic response. Eosinophils are often implicated in allergic diseases, especially in contribution to tissue injury, by releasing cytotoxic granule proteins [32], although GPR120 activation did not appear to induce degranulation in terms of EDN release. Likewise, IL-5-mediated eosinophil survival, a key mechanism involved in eosinophilic inflammation, was not influenced by GPR120 stimulation. Our results appear to indicate that GPR120 maintains the resting eosinophils in physiological conditions rather than activating eosinophils in the pathological conditions.

Without the survival factors, isolated eosinophils spontaneously undergo apoptosis. Here we have provided multiple lines of evidence showing the pro-survival effects of GPR120 on eosinophil apoptosis. GW9508 dramatically suppressed the number of eosinophils that underwent apoptosis and membrane Fas receptor expression (Fig. 4). GW9508-induced eosinophil survival was inhibited by pharmacological PI3K inhibitors, indicating PI3K signaling was involved in the anti-apoptotic effect. Caspases, a family of cysteine proteases, are activated in the interior of cells during the process to apoptosis. The spontaneous increase in caspase-3 activities normally observed upon cell culture was diminished in GW9508-stimulated cells. Several studies have implicated GPR120 in inhibition of apoptosis. In pancreatic islets, GPR120 activation prevented palmitate- and linoleic acid-induced apoptosis [33]. Consistent with our data, a GPR120-dependent anti-apoptotic effect and inhibition of caspase-3 activities have been reported in enteroendocrine cells [34].

We previously reported that a natural GPR120 ligand, DHA, promoted eosinophil apoptosis and inhibited CCR3-driven migration [23]. Although DHA’s effects on eosinophils were contrary to those of GW9508 observed in the current study, it may not be surprising due to the multiple molecular targets of FFA. Moreover, human eosinophils contain abundant amounts of 15-lipoxigenase, which can rapidly convert DHA into anti-inflammatory mediators such as protectin D1 [35].

Recent evidence highlights the critical role of adipose tissue-resident eosinophils in regulating metabolic homeostasis. Wu *et al*. demonstrated that eosinophils were the major IL-4-expressing cells in adipose tissue, and that their absence greatly attenuated the alternatively activated macrophages that are necessary to maintain glucose homeostasis [5]. Another study reported that cold exposure or exercise induces the secretion of meteorin-like, a peptide that triggers the production of IL-4 by eosinophils in adipose tissue [7]. IL-4-stimulated alternative macrophages induce the production of catecholamines that increase brown fat thermogenesis. In this context, we measured IL-4 production induced by a GPR120 agonist using ELISpot assay. As shown in Fig. 5, we were able to show the significant release of IL-4 in GW9508-stimulated eosinophils. Additional studies are required to elucidate the physiological relevance of GPR120 expressed in eosinophils, although our results are potentially important for understanding eosinophils in regulating energy metabolism.

Interestingly, eosinophils themselves are functionally regulated by various receptors that can sense the extracellular environment affected by nutrients. For instance, human eosinophils express the receptor for adipocyte-derived cytokines (adipocytokines). Leptin positively regulates eosinophil chemotaxis and cytokine secretion [36,37] while adiponectin inhibits chemokine-induced eosinophil migration and adhesion [12]. Metabolite-sensing nuclear hormone receptors also regulate eosinophil functions. Vitamin A metabolites and retinoic acids are very potent survival factors of eosinophils through retinoic acid receptors (RARs) and RXR [14]. Taken together with current data, these findings provide the evidence for the previously unrecognized mechanisms underlying the association between marked changes in dietary consumption patterns and the immune system.

In summary, we have characterized the expression and functions of fatty acid sensor GPR120 on human eosinophils by *in vitro* experiments using pharmacological agonists. To the best of our knowledge, this is the first demonstration of functional GPR120 expression on eosinophils. GPR120 agonists could suppress cytokine-deprived spontaneous apoptosis, which is associate with down-regulation of Fas receptor expression. GPR120 agonist-induced eosinophil survival was likely mediated through the PI3K signaling and inhibition of caspase-3 activity. Furthermore, GPR120 agonist-stimulated eosinophils release significant amounts of IL-4. Eosinophils in adipose tissue and the gastrointestinal tract where they normally reside might be sensing extracellular FFAs through GPR120 and regulate their longevity and local immune responses.

## Supporting Information

S1 Fig The effect of GW9508 was not mediated through GPR40. (A) GPR40 expression was not detected on human eosinophils. Cells were fixed and permeabilized, and then stained with anti-GPR40 antibody (open histogram) or isotype-matched control (filled histogram), followed by flow cytometric analysis. A HeLa cell line was used as a positive control. Representative results are shown. (B) GW1100, a GPR40-specific antagonist, did not affect GW9508-induced eosinophil survival. Cells were preincubated with GW1100 (10 μM) for 30 min, followed by treatment with or without GW9508 (100 μM) for 48 h. The percentage of live cells (Annexin V- and PI-negative cells) was measured and the data are expressed as the mean ± SEM (n = 3). n.s: not significant. (C) GW1100 did not affect GW9508-induced eosinophil IL-4 secretion. Cells were preincubated with GW1100 (10 μM) for 30 min, followed by treatment with or without GW9508 (100 μM) for 18 h. IL-4 ELISpot assay was performed, and the developed spots were counted by a single investigator in a coded manner. The data are expressed as the mean ± SEM (n = 4). n.s: not significant.
(TIF)

S2 Fig GPR120 agonist did not affect eosinophil chemotaxis and induce degranulation. (A) Chemotactic response toward GW9508 was assessed by Boyden chambers, although no significant effect was observed. Data are expressed as the mean of three experiments ± SEM from different donors. (B) Eosinophils were pretreated with or without the indicated concentrations of GW9508 for 60 min, and then eotaxin-induced chemotaxis assays were performed. No significant effect was observed as a result of pretreatment with GW9508. Data are expressed as the mean of four experiments ± SEM from different donors. (C) After incubation with the indicated concentration of GW9508 for 4 h, the EDN concentration in the culture supernatants was measured by ELISA. No significant effect was observed. Data are expressed as the mean of five experiments ± SEM from different donors.
(TIF)

## References

[1] Shamri R, Xenakis JJ, Spencer LA (2011) Eosinophils in innate immunity: an evolving story. Cell Tissue Res 343: 57–83. 10.1007/s00441-010-1049-6 21042920 PMC3679536

[2] Giembycz MA, Lindsay MA (1999) Pharmacology of the eosinophil. Pharmacol Rev 51: 213–340. 10353986

[3] Rothenberg ME, Hogan SP (2006) The eosinophil. Annu Rev Immunol 24: 147–174. 16551246 10.1146/annurev.immunol.24.021605.090720

[4] Lloyd CM, Saglani S (2013) Eosinophils in the spotlight: Finding the link between obesity and asthma. Nat Med 19: 976–977. 10.1038/nm.3296 23921744

[5] Wu D, Molofsky AB, Liang HE, Ricardo-Gonzalez RR, Jouihan HA, et al (2011) Eosinophils sustain adipose alternatively activated macrophages associated with glucose homeostasis. Science 332: 243–247. 10.1126/science.1201475 21436399 PMC3144160

[6] Molofsky AB, Nussbaum JC, Liang HE, Van Dyken SJ, Cheng LE, et al (2013) Innate lymphoid type 2 cells sustain visceral adipose tissue eosinophils and alternatively activated macrophages. J Exp Med 210: 535–549. 10.1084/jem.20121964 23420878 PMC3600903

[7] Rao RR, Long JZ, White JP, Svensson KJ, Lou J, et al (2014) Meteorin-like is a hormone that regulates immune-adipose interactions to increase beige fat thermogenesis. Cell 157: 1279–1291. 10.1016/j.cell.2014.03.065 24906147 PMC4131287

[8] Fredriksson R, Hoglund PJ, Gloriam DE, Lagerstrom MC, Schioth HB (2003) Seven evolutionarily conserved human rhodopsin G protein-coupled receptors lacking close relatives. FEBS Lett 554: 381–388. 14623098 10.1016/s0014-5793(03)01196-7

[9] Hirasawa A, Tsumaya K, Awaji T, Katsuma S, Adachi T, et al (2005) Free fatty acids regulate gut incretin glucagon-like peptide-1 secretion through GPR120. Nat Med 11: 90–94. 15619630 10.1038/nm1168

[10] Oh DY, Talukdar S, Bae EJ, Imamura T, Morinaga H, et al (2010) GPR120 is an omega-3 fatty acid receptor mediating potent anti-inflammatory and insulin-sensitizing effects. Cell 142: 687–698. 10.1016/j.cell.2010.07.041 20813258 PMC2956412

[11] Ichimura A, Hirasawa A, Poulain-Godefroy O, Bonnefond A, Hara T, et al (2012) Dysfunction of lipid sensor GPR120 leads to obesity in both mouse and human. Nature 483: 350–354. 10.1038/nature10798 22343897

[12] Yamamoto R, Ueki S, Moritoki Y, Kobayashi Y, Oyamada H, et al. (2013) Adiponectin attenuates human eosinophil adhesion and chemotaxis: implications in allergic inflammation. J Asthma. 10.3109/02770903.2013.816725 23777560

[13] Ueki S, Adachi T, Bourdeaux J, Oyamada H, Yamada Y, et al (2003) Expression of PPARgamma in eosinophils and its functional role in survival and chemotaxis. Immunol Lett 86: 183–189. 12644321 10.1016/s0165-2478(03)00003-8

[14] Ueki S, Mahemuti G, Oyamada H, Kato H, Kihara J, et al (2008) Retinoic acids are potent inhibitors of spontaneous human eosinophil apoptosis. J Immunol 181: 7689–7698. 19017957 10.4049/jimmunol.181.11.7689

[15] Kobayashi Y, Ueki S, Mahemuti G, Chiba T, Oyamada H, et al (2005) Physiological levels of 15-deoxy-Delta12,14-prostaglandin J2 prime eotaxin-induced chemotaxis on human eosinophils through peroxisome proliferator-activated receptor-gamma ligation. J Immunol 175: 5744–5750. 16237065 10.4049/jimmunol.175.9.5744

[16] Matsuwaki Y, Ueki S, Adachi T, Oyamada H, Kamada Y, et al (2005) The synthetic PPARgamma agonist troglitazone inhibits IL-5-induced CD69 upregulation and eosinophil-derived neurotoxin release from eosinophils. Pharmacology 74: 169–173. 15818060 10.1159/000085034

[17] Adachi T, Choudhury BK, Stafford S, Sur S, Alam R (2000) The differential role of extracellular signal-regulated kinases and p38 mitogen-activated protein kinase in eosinophil functions. J Immunol 165: 2198–2204. 10925307 10.4049/jimmunol.165.4.2198

[18] Briscoe CP, Peat AJ, McKeown SC, Corbett DF, Goetz AS, et al (2006) Pharmacological regulation of insulin secretion in MIN6 cells through the fatty acid receptor GPR40: identification of agonist and antagonist small molecules. Br J Pharmacol 148: 619–628. 16702987 10.1038/sj.bjp.0706770 PMC1751878

[19] Tamaki M, Konno Y, Kobayashi Y, Takeda M, Itoga M, et al (2014) Expression and functional roles of G-protein-coupled estrogen receptor (GPER) in human eosinophils. Immunol Lett 160: 72–78. 10.1016/j.imlet.2014.03.012 24718279

[20] Saito Y, Takeda M, Nishikawa J, Konno Y, Tamaki M, et al (2014) The effect of pharmacological PI3Kgamma inhibitor on eotaxin-induced human eosinophil functions. Pulm Pharmacol Ther 27: 164–169. 10.1016/j.pupt.2013.11.006 24333185

[21] Bandeira-Melo C, Perez SA, Melo RC, Ghiran I, Weller PF (2003) EliCell assay for the detection of released cytokines from eosinophils. J Immunol Methods 276: 227–237. 12738376 10.1016/s0022-1759(03)00076-0

[22] Druilhe A, Cai Z, Haile S, Chouaib S, Pretolani M (1996) Fas-mediated apoptosis in cultured human eosinophils. Blood 87: 2822–2830. 8639900

[23] Tanigai T, Ueki S, Kihara J, Kamada R, Yamauchi Y, et al (2012) Docosahexaenoic acid exerts anti-inflammatory action on human eosinophils through peroxisome proliferator-activated receptor-independent mechanisms. Int Arch Allergy Immunol 158: 375–386. 10.1159/000332965 22487606

[24] Wu Q, Wang H, Zhao X, Shi Y, Jin M, et al (2013) Identification of G-protein-coupled receptor 120 as a tumor-promoting receptor that induces angiogenesis and migration in human colorectal carcinoma. Oncogene 32: 5541–5550. 10.1038/onc.2013.264 23851494

[25] Carvalheira JB, Qiu Y, Chawla A (2013) Blood spotlight on leukocytes and obesity. Blood 122: 3263–3267. 10.1182/blood-2013-04-459446 24065242 PMC3821723

[26] Sanchez-Reyes OB, Romero-Avila MT, Castillo-Badillo JA, Takei Y, Hirasawa A, et al (2014) Free fatty acids and protein kinase C activation induce GPR120 (free fatty acid receptor 4) phosphorylation. Eur J Pharmacol 723: 368–374. 10.1016/j.ejphar.2013.11.003 24239485

[27] Talukdar S, Olefsky JM, Osborn O (2011) Targeting GPR120 and other fatty acid-sensing GPCRs ameliorates insulin resistance and inflammatory diseases. Trends Pharmacol Sci 32: 543–550. 10.1016/j.tips.2011.04.004 21663979 PMC3419590

[28] Hudson BD, Shimpukade B, Mackenzie AE, Butcher AJ, Pediani JD, et al (2013) The pharmacology of TUG-891, a potent and selective agonist of the free fatty acid receptor 4 (FFA4/GPR120), demonstrates both potential opportunity and possible challenges to therapeutic agonism. Mol Pharmacol 84: 710–725. 10.1124/mol.113.087783 23979972 PMC3807074

[29] Cartoni C, Yasumatsu K, Ohkuri T, Shigemura N, Yoshida R, et al (2010) Taste preference for fatty acids is mediated by GPR40 and GPR120. J Neurosci 30: 8376–8382. 10.1523/JNEUROSCI.0496-10.2010 20573884 PMC6634626

[30] Watson SJ, Brown AJ, Holliday ND (2012) Differential signaling by splice variants of the human free fatty acid receptor GPR120. Mol Pharmacol 81: 631–642. 10.1124/mol.111.077388 22282525 PMC3336805

[31] Woerly G, Honda K, Loyens M, Papin JP, Auwerx J, et al (2003) Peroxisome proliferator-activated receptors alpha and gamma down-regulate allergic inflammation and eosinophil activation. J Exp Med 198: 411–421. 12900517 10.1084/jem.20021384 PMC2194090

[32] Kita H, Weiler DA, Abu-Ghazaleh R, Sanderson CJ, Gleich GJ (1992) Release of granule proteins from eosinophils cultured with IL-5. J Immunol 149: 629–635. 1624806

[33] Taneera J, Lang S, Sharma A, Fadista J, Zhou Y, et al (2012) A systems genetics approach identifies genes and pathways for type 2 diabetes in human islets. Cell Metab 16: 122–134. 10.1016/j.cmet.2012.06.006 22768844

[34] Katsuma S, Hatae N, Yano T, Ruike Y, Kimura M, et al (2005) Free fatty acids inhibit serum deprivation-induced apoptosis through GPR120 in a murine enteroendocrine cell line STC-1. J Biol Chem 280: 19507–19515. 15774482 10.1074/jbc.M412385200

[35] Miyata J, Fukunaga K, Iwamoto R, Isobe Y, Niimi K, et al (2013) Dysregulated synthesis of protectin D1 in eosinophils from patients with severe asthma. J Allergy Clin Immunol 131: 353–360 e351–352. 10.1016/j.jaci.2012.07.048 23006546

[36] Kato H, Ueki S, Kamada R, Kihara J, Yamauchi Y, et al (2011) Leptin has a priming effect on eotaxin-induced human eosinophil chemotaxis. Int Arch Allergy Immunol 155: 335–344. 10.1159/000321195 21346363

[37] Wong CK, Cheung PF, Lam CW (2007) Leptin-mediated cytokine release and migration of eosinophils: implications for immunopathophysiology of allergic inflammation. Eur J Immunol 37: 2337–2348. 17634954 10.1002/eji.200636866

