# Correction: Functional Analysis of Free Fatty Acid Receptor GPR120 in Human Eosinophils: Implications in Metabolic Homeostasis

**DOI:** 10.1371/journal.pone.0157208

**Published:** 2016-06-03

**Authors:** Yasunori Konno, Shigeharu Ueki, Masahide Takeda, Yoshiki Kobayashi, Mami Tamaki, Yuki Moritoki, Hajime Oyamada, Masamichi Itoga, Hiroyuki Kayaba, Ayumi Omokawa, Makoto Hirokawa

In the Materials and Methods section, there is an error in the second and third sentences of the section titled “Flow cytometric analysis for receptor expression.” The correct sentence is: Cells were then stained with anti-human GPR120 mAb (1:50, Santa Cruz Biotechnology) or isotype-matched control mAb (Santa Cruz Biotechnology) for 30 min on ice. Next, the cells were incubated with Alexa Fluor 488 anti-goat IgG (1:100, Life Technologies Corporation) for 30 min on ice.
