# Supplementary figures and images for: Functional Analysis of Free Fatty Acid Receptor GPR120 in Human Eosinophils: Implications in Metabolic Homeostasis
Source: PLoS One. 2015 Mar 19;10(3):e0120386. doi: 10.1371/journal.pone.0120386 (PMC4366258; doi:10.1371/journal.pone.0120386)

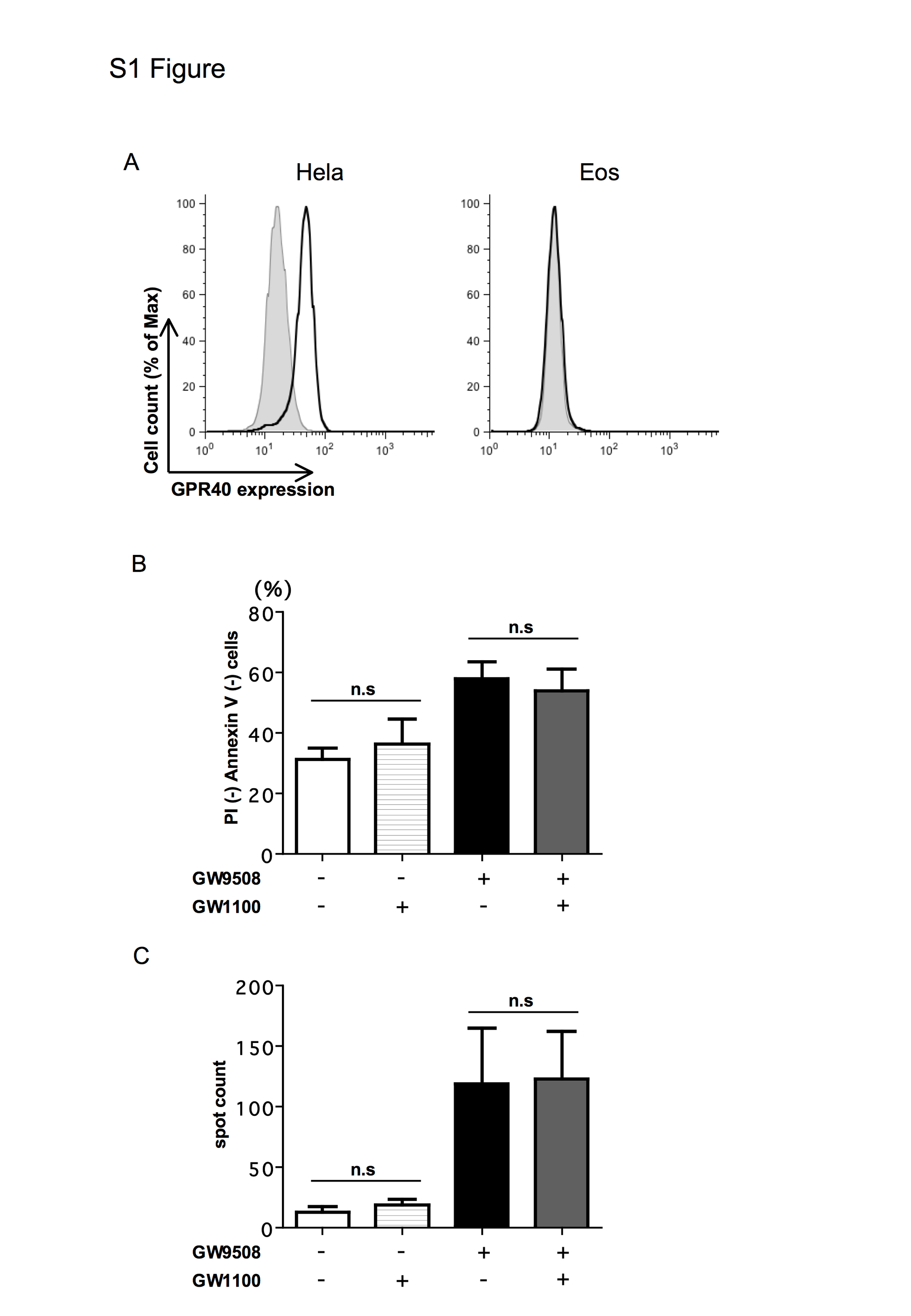

Supplement: S1 Fig — (A) GPR40 expression was not detected on human eosinophils. Cells were fixed and permeabilized, and then stained with anti-GPR40 antibody (open histogram) or isotype-matched control (filled histogram), followed by flow cytometric analysis. A HeLa cell line was used as a positive control. Representative results are shown. (B) GW1100, a GPR40-specific antagonist, did not affect GW9508-induced eosinophil survival. Cells were preincubated with GW1100 (10 μM) for 30 min, followed by treatment with or without GW9508 (100 μM) for 48 h. The percentage of live cells (Annexin V- and PI-negative cells) was measured and the data are expressed as the mean ± SEM (n = 3). n.s: not significant. (C) GW1100 did not affect GW9508-induced eosinophil IL-4 secretion. Cells were preincubated with GW1100 (10 μM) for 30 min, followed by treatment with or without GW9508 (100 μM) for 18 h. IL-4 ELISpot assay was performed, and the developed spots were counted by a single investigator in a coded manner. The data are expressed as the mean ± SEM (n = 4). n.s: not significant. (TIF) [file pone.0120386.s001.tif]

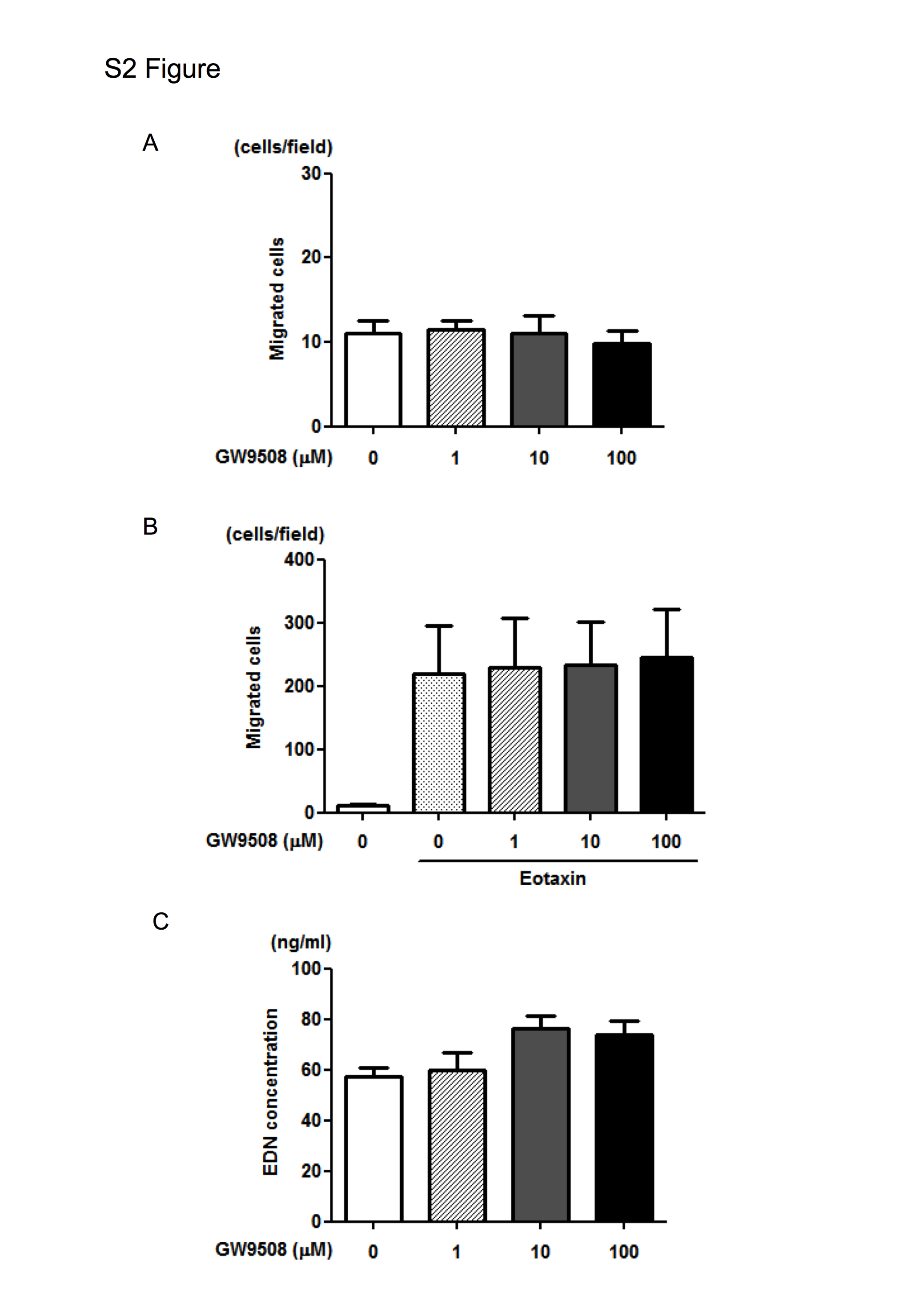

Supplement: S2 Fig — (A) Chemotactic response toward GW9508 was assessed by Boyden chambers, although no significant effect was observed. Data are expressed as the mean of three experiments ± SEM from different donors. (B) Eosinophils were pretreated with or without the indicated concentrations of GW9508 for 60 min, and then eotaxin-induced chemotaxis assays were performed. No significant effect was observed as a result of pretreatment with GW9508. Data are expressed as the mean of four experiments ± SEM from different donors. (C) After incubation with the indicated concentration of GW9508 for 4 h, the EDN concentration in the culture supernatants was measured by ELISA. No significant effect was observed. Data are expressed as the mean of five experiments ± SEM from different donors. (TIF) [file pone.0120386.s002.tif]
